# Gambling disorder comorbidity a narrative review

**DOI:** 10.1080/19585969.2025.2484288

**Published:** 2025-04-03

**Authors:** Rishi Sharma, Aviv Weinstein

**Affiliations:** Department of Psychology, Ariel University, Ariel, Israel

**Keywords:** Gambling disorder, problematic gambling, comorbidity, alcohol and drug use disorder, mood disorders, anxiety

## Abstract

**Introduction:** Problematic and pathological gambling (PG) lead to major adverse consequences for individuals, their families, and society and is highly comorbid with numerous other mental health disorders.

**Methods:** This narrative review summarized population-based, cross-sectional, treatment and prospective studies on gambling disorder and comorbidity over the past 14 years.

**Results:** These studies show a high rate of comorbidity of PG and substance and alcohol use disorders, mood and anxiety disorders. Prospective studies indicate that, in some cases, gambling precedes the onset of the comorbid disorder, while in other instances, the temporal relationship is reversed. Women face greater psychiatric comorbidity and are more likely to have mood disorders, suicidality, mania, anxiety and alcohol dependence. Treatment of PG can be effective by improving the gambling and depressive symptoms of PG.

**Conclusions**: Over the past 15 years, significant progress has been made in understanding and treating GD and its psychiatric comorbidities, with evidence highlighting the reciprocal relationships between GD and conditions like substance use, mood and anxiety disorders.

## Introduction

Gambling disorder (GD), according to the Diagnostic and Statistical Manual of Mental Disorders, Fifth Edition (DSM-5) (American Psychiatric Association 2013), is characterised by persistent and recurrent problematic gambling behaviour that leads to significant adverse effects on the individual, the family, and society. Adults and adolescents with GD struggle to control their gambling activities, leading them to persist in gambling despite negative consequences.

Prior to the DSM-V the term ‘Pathological gambling’ was used in DSM-III and DSM-IV as an impulse-control disorder being persistent and recurrent maladaptive gambling behaviour and this term was being phased out in research due to its stigmatising nature. Gambling disorder in the DSM-5 is the only non-substance addiction in the new category of ‘Addiction and Related Disorders’ substance addiction due to commonalities between GD and substance use disorders (SUD) (Hodgins et al. 2011; Petry et al. 2014). Over the past 15 years, there has been a substantial surge in commercial gambling opportunities, resulting in a notable increase in the prevalence of GD associated comorbidities, and other gambling-related harms. The growth of commercial gambling has been unprecedented, with global gambling losses estimated to have reached a staggering $540 billion in 2023 (European Business Magazine 2024).

Gambling and substance use share many features. Individuals with GD and SUD report that this behaviour is rewarding and exciting, and that they have an inability to control this behaviour despite its harmful consequences, including illegal, financial or social consequences. Both GD and SUD are characterised by an impulsive risky behaviour which is done without prior thinking, and it results in harmful long-term consequences. The impulsivity that underlies GD and SUD appears during late adolescence or early adulthood when the brain is undergoing a maturation process. Finally, GD like SUD is related to worse academic performance, anxiety, and poorer quality of life which in turn can interfere with career goals and social relationships (Grant and Chamberlain 2020).

During the past 15 years, there has been an increase in gambling research, particularly on GD. Short and long-term treatment interventions involving both behavioural and pharmacological approaches have been developed; however, many suffer from methodo­logical limitations, and few effective studies are conducted in clinical settings. Behavioural interventions, particularly cognitive-behavioural therapy (CBT) but also motivational interviewing and Gamblers Anonymous, are used in the treatment of gambling disorders. No pharmacological therapy has a formal indication for the treatment of gambling disorder, although placebo-controlled trials suggest that some medications, such as opioid-receptor antagonists, may be helpful (Yip and Potenza 2014; Potenza et al. 2019).

The DSM-V allows for the distinction between episodic and continuous variants of GD, as well as for the disorder to be classified as in early or sustained remission. A recent meta-analysis found a prevalence of moderate risk/at-risk gambling to be 2.43% and of problem/pathological gambling to be 1.29% in the adult population (Gabellini et al. 2023). Gambling research has found that there is a large number of individuals with low-risk and moderate-risk for gambling who also experience harm. Although these groups compromise a relatively small percentage of the population, their numbers are greater than those identified as suffering from GD and they experience greater harm than individuals with GD (Petry et al. 2005). Population-based studies like the National Epidemiologic Survey on Alcohol and Related Conditions (NESARC) study by Hilbrecht and Mock (2019) found that pathological gamblers had a six-fold increased risk of a lifetime alcohol misuse diagnosis and a 4.4 times higher risk of a substance use disorder compared with non-gamblers. They were also about a 3 times higher probability of having major depression or dysthymia and an 8 times higher probability of having experienced a manic episode. Rates of generalised anxiety disorder, panic disorder, and specific phobias were over three times higher in gamblers, while social phobia was twice as prevalent. Pathological gamblers also had an eightfold increased risk of personality disorders. Modules for obsessive-compulsive and post-traumatic stress disorders were not included, and evidence for their comorbidity with pathological gambling remains unclear. The National Comorbidity Survey Replication (NCS-R), a major US study, revealed significant comorbidity between pathological gambling and other psychiatric disorders. Pathological gamblers showed higher risks for substance use (5.5 times), mood (3.7 times), and anxiety disorders (3.1 times). The NCS-R, pioneering in collecting retrospective onset data, found that in most cases (74.3%), pathological gambling followed the comorbid disorder. Mood and anxiety disorders often preceded pathological gambling, while pathological gambling more frequently preceded substance use disorders. These findings highlight a strong association between pathological gambling, substance use, mood, and anxiety disorders, although the exact nature of these relationships requires further study (Kessler et al. 2008). Since the NCS-R study in 2005, there were new studies describing comorbidity of GD with other mental disorders. By synthesising existing literature, the review explores shared risk factors such as impulsivity, trauma, and sociodemographic influence, as well as treatment approaches.

## Methodology

### Search strategy

**Figure 1.**
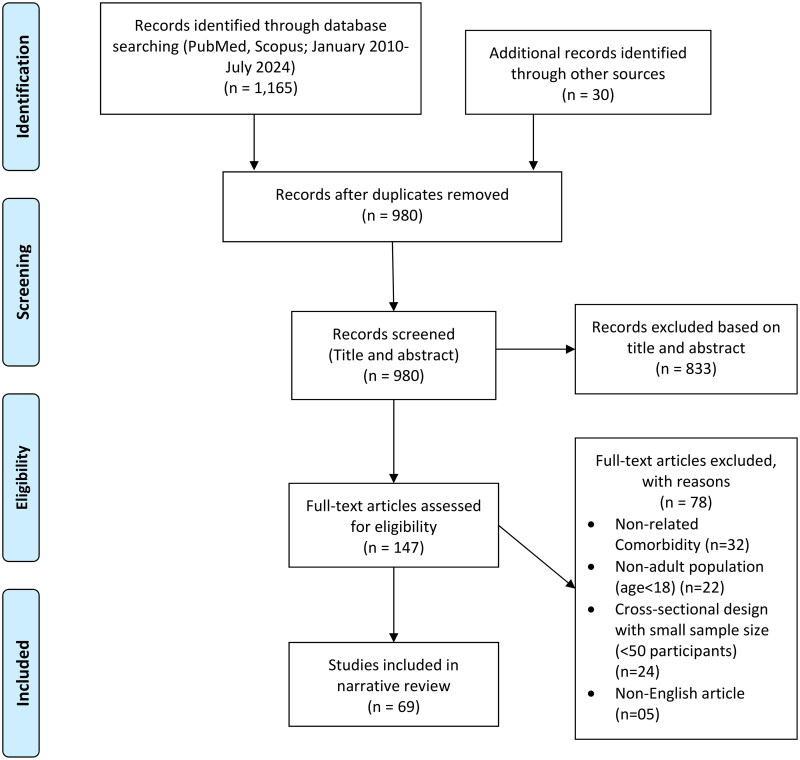
Study selection flowchart.

In view of the growing research on PG over the past 15 years, this narrative review involved a systematic search in PubMed and Scopus databases for studies on gambling disorder and its psychiatric comorbidities from January 2010 and July 2024, using the keywords variations of ‘gambling disorders’, ‘problematic gambling’, ‘pathological gambling’ combined with terms for psychiatric comorbidities such as ‘substance use disorder’, ‘alcohol use disorder’, ‘depression’, ‘mood disorder’, ‘anxiety disorder’, ‘personality disorder’ and ‘ADHD’. Filters were applied to include only English-language articles involving adult and young populations (ages 18+) with a focus on longitudinal and cohort studies to highlight the progression and interactions of GD and psychiatric conditions over time. Titles and abstracts were initially screened, followed by a full-text review to confirm relevance and adherence to inclusion criteria. Studies with adolescents or small samples (<50 participants) and non-peer-reviewed sources were excluded. A total of 49 studies, including 29 studies on comorbidity with substance and alcohol use disorders and 20 studies on affective and anxiety disorders, were selected for the final review, with additional relevant studies identified through reference lists of included studies. Although there are other psychiatric comorbidities associated with GD (such as Obsessive Compulsive Disorder-OCD, conduct disorders, Internet and Gaming Disorder IGD, Compulsive Sexual Behaviour-CSB, Compulsive Buying CB and eating disorders), we have excluded them from the review due the paucity of evidence and limitation of the scope of the review. PRISMA style flowchart tailored to narrative review described in Figure 1.

## Results

### Drug and alcohol use disorders

#### Population-based studies

Population studies include studies analysing general population trends, such as large-scale epidemiological or national survey data. Bischof et al. (2013) include a random sample of German adult gamblers (*N* = 15,023), where high rates of psychiatric comorbidity, particularly substance use disorders, were observed, with lifetime prevalence rates of 93.6% for pathological gamblers, 83.5% for problem gamblers, and 81% for at-risk gamblers.

A national survey (*N* = 2,963) from 2011 to 2013 identified gambling as the most prevalent addictive behaviour, followed by alcohol use, tobacco use and marijuana use (Barnes et al. 2015). A study on Swedish participants (*N* = 2,099) found associations between gambling and substance use, anxiety, and affective disorders, with women showing higher rates of anxiety and affective disorders (Karlsson and Håkansson 2018). Kotyuk et al. (2020) reported adolescents and young adults (min age-18; *n* = 3,003) showed that gambling severity was linked to smoking, alcohol, and cannabis use. The NESARC data (*N* = 43,093) demonstrated that cannabis use influenced the relationships between problem gambling severity and psychopathology, particularly major depression, paranoid personality disorder, and antisocial personality disorder (Hammond et al. 2020). A longitudinal study using the Norwegian national patient registry (*N* = 5131) found that individuals with prior substance use disorders were more likely to be subsequently diagnosed with GD (Girard et al. 2023). Among Canadian gamblers (*N* = 10,054), a significant proportion of cannabis users reported smoking cannabis while gambling (McGrath et al. 2023).

#### Specific population studies

A separate study focusing on US veterans (*N* = 3,157) found that at-risk and problem gambling was associated with higher rates of substance use, anxiety and depressive disorders and physical or sexual trauma (Stefanovics et al. 2017). In a study of community gamblers (*N* = 562), individuals with problematic cocaine use (9.3%) were more likely to be pathological gamblers, presenting with increased impulsivity, depression, anxiety, stress, and alcohol consumption compared to recreational cocaine users or non-users (Ethier et al. 2020). In a study of 1.1 million Veterans Affairs (VA) mental health service users, pathological gambling was associated with increased substance use, bipolar, anxiety and personality disorders (Edens and Rosenheck 2012). Veterans with pathological gambling had higher rates of PTSD, depression, and anxiety. Among people with HIV, increased odds for GD were linked to male gender, African American race, substance use and anxiety disorders (Langan et al. 2019). A Swedish case-control study (*N* = 3,592) found that women with gambling disorders appeared to be at higher risk of psychiatric comorbidity and socioeconomic vulnerability compared to men, with women more likely to have their psychiatric and psychosocial problems diagnosed before gambling disorder, while men more often received concurrent diagnoses (Larsson and Håkansson 2022). Approximately 50% of individuals with gambling disorder also experienced problems with alcohol, illicit drugs, and prescription sedatives or painkillers, indicating severe distress and substance use (André et al. 2021). Predictors of cannabis use among gamblers included greater problem gambling severity, more hours spent gambling, a larger range of gambling activities, tobacco use, and significant child abuse. Additionally, gambling and problem gambling were associated with tobacco use and heavier smoking among 651 patients from residential SUD treatment programs, suggesting that individuals with comorbid SUD and GD may need concurrent treatment for both tobacco use and problem gambling (Pagano et al. 2023).

*In summary*, gender disparity exists in gambling disorder, with women potentially facing greater psychiatric comorbidity and socioeconomic vulnerability, while frequent online gambling and cannabis use are linked with greater gambling severity, highlighting the need for comprehensive treatment approaches.

Table 1 shows studies of comorbidity of Gambling disorder with alcohol and substance use disorders.

**Table 1.** Gambling disorder and substance and alcohol use disorder.

Study	Country	Study type	Sample Size (n)	Healthy control	Problem/Pathological Gambling Disorder Diagnostic Tool	Associated comorbidity	Comorbidity Diagnostic Tool	No. of Problem / Pathological Gamblers in samples	Key findings	Ref
Hodgins and El-Guebaly 2010	Canada	Longitudinal	101	No	SOGS	Substance dependence, mood disorder	AUDADIS-IV	101	Substance dependence and mood disorders were associated with poorer long-term outcomes for pathological gamblers, emphasising the importance of addressing comorbidities in treatment.	Leino et al. 2023
Chou and Afifi 2011	United States	Longitudinal	33231	No	DSM-IV criteria	Axis I psychiatric disorders, SUD, AUD, Alcohol dependence disorder	AUDA DIS-IV	209	Disordered gambling is a risk factor for subsequent Axis I disorders (mood, anxiety, PTSD, substance use, alcohol use, alcohol dependence) after adjusting for demographics, health factors, and stressful life events.	Szerman et al. 2023
Edens and Rosenheck 2012	United States	Cross-sectional	1,060,148	No	SOGS	Depression, PTSD, Alcohol Dependence	AUDADIS-IV	2198	Past-year rate of PG diagnosis among veterans treated in specialty MH program was 0.2%, significantly lower than prevalence rates in other treatment samples and the general U.S population.	Stefanovics et al. 2017
Pilver et al. 2013	USA	Longitudinal	10,231	No	DSM-IV Gambling Disorder criteria	Axis I disorders (Mood, anxiety, SUD)	AUDADIS-IV	269	Individuals with past-year problem gambling were more likely to develop new cases of mood disorders, anxiety disorders, and substance use disorders compared to those with no/low-risk gambling.	Hodgins and El-Guebaly 2010
Bischof et al. 2013	Germany	Cross-sectional	164 (gambling group)	Yes (general population, n = 15023)	DSM-IV	SUD, Mood disorders, Anxiety disorders	CIDI	164	Individuals with pathological, problem, or at-risk gambling had higher rates of comorbid Axis I disorders, particularly mood and anxiety disorders.	Hilbrecht and Mock 2019
Parhami et al. 2014	USA	Longitudinal	34,653	No	DSM-IV	Mood, anxiety, personality disorders and SUD	AUDADIS-IV	141	Baseline recreational gambling was associated with increased incidence of mood disorders, generalised anxiety disorder, and substance use disorders at follow-up	Chou and Afifi 2011
Soberay et al. 2014	USA	Cross-sectional study	71	No	NODS	Depression, Bipolar, Anxiety Disorder, PTSD	HANDS Depression Screen, MDQ, Carroll-Davidson GAD Screen, SPRINT-4 PTSD	53	- More co-occurring disorders linked to greater gambling severity. - Lower psychosocial functioning at treatment starts with more co-occurring disorders. - No impact of co-occurring disorders on treatment progress or satisfaction.	André et al. 2021
Cowlishaw and Hakes 2015	United States	Longitudinal	43,093	No	NODS	Substance use disorder	AUDADIS-IV	402	Problem gambling is elevated in substance use treatment but less common than previously thought.	Pagano et al. 2023
Barnes et al. 2015	United States	Cross-sectional	2,963	No	NODS	Alcohol abuse/dependence, Tobacco dependence, Marijuana abuse/dependence	AUDADIS-IV	Not mentioned	- Past-year gambling was most prevalent (76.9%) followed by alcohol (67.6%), tobacco (28.7%), and marijuana use (11.2%). - Problem gambling was associated with all three substance use measures. - Male, Black, and lower socioeconomic status individuals were more likely to have problem gambling and co-occurring substance use disorders.	Kessler et al. 2008
Himelhoch et al. 2016	United States	Cross-sectional	185	No	DSM-5	Substance use disorder	--	85 (45.9%)	High prevalence of gambling disorder (46.2%) among people attending methadone maintenance treatment program	Soberay et al. 2014
Davis et al. 2017	United States	Cross-sectional	833	No	ASSIST	Binge drinking; SUD	AUDADIS-IV	288 (34.3%)	Binge drinking and non-partner aggression were associated with gambling among Veterans receiving substance use treatment.	Carr et al. 2021
Mann et al. 2017	Germany	Case-control	515	Yes (n = 269)	SOGS DSM-IV	Substance dependence (88%), nicotine dependence (80%), alcohol dependence (28%)	SCID-I, NEO-FFI, FTND, BIS	515	PG had higher rates of comorbid psychiatric disorders, positive family history of addiction, and higher levels of neuroticism and impulsivity compared to healthy controls.	Silbernagl et al. 2019
Stefanovics et al. 2017	USA	Cross-sectional	3,157	No	BBGS, SOGS	Psychiatry disorders and suicidality	PCL-S, PHQ-4, MINI, FTND, PSQI	Recreational gambling, n = 1095; At risk/Problem and pathological gambling, n = 57	Veterans with problem gambling have higher rates of mental health disorders	Girard et al. 2023
Karlsson and Håkansson 2018	Sweden	Longitudinal National registry	2,099	No	ICD-10	SUD, mood, anxiety and personality disorder, mortality & suicide rates	ICD-10		Mortality and suicide rates are significantly elevated among individuals with GD; depression predicts suicide death.	Bischof et al. 2013
Bergamini et al. 2018	Italy	Cross-sectional	900	No	CPGI	Psychiatric comorbidities, Drug abuse/dependence, Tobacco smoking	MINI	47 (5.3% of total sample)	- In those who gambled over the last year, 10.1% were at-risk gamblers. - At-risk gambling preceded the onset of a major psychiatric disorder in 52.1% of cases.	Mann et al. 2017
Langan et al. 2019	United States	Cross-sectional	100	No	BBGS	Alcohol use disorder, Marijuana and Heroin use, Impulsivity	DSM-5	13	− 13% met criteria for DSM-5 GD - Problem gamblers more likely to report marijuana and heroin use, and higher impulsivity	Ethier et al. 2020
Hammond et al. 2020	United States	Longitudinal	43,093 NESARC study	No	DSM-IV	Cannabis use, psychiatric disorder	AUDADIS-IV	1,265	Cannabis use was associated with more severe problem gambling and increased psychiatric comorbidity.	Karlsson and Håkansson 2018
Silbernagl et al. 2019	Austria	Cross-sectional	355	No	DSM IV	ADHD, Major Depressive Disorder, Antisocial Personality Disorder	MINI, Adult ADHD self-report scale, Wender Utah Rating Scale	80	Patients with problem gambling had higher rates of psychiatric comorbidities, particularly depression. ADHD predicted membership in the class with the most severe comorbidity burden.	Cowlishaw and Hakes 2015
Ethier et al. 2020	Canada	Cross-sectional	562	No	PGSI, GMQ-F	Psychological, SUD and Personality disorder	ASSIST-V, AUDIT-C, FTND	51	Problematic cocaine use is more prevalent among gamblers than non-gamblers	McGrath et al. 2023
Kotyuk et al. 2020	Hungary	Cross sectional	3003	No	POGQ-SF	Smoking, gambling, alcohol and cannabis consumption	BSMAS, SCOFF, EAI, MGH-HPS, PIUQ	50	High comorbidity between problem gambling and other addictive behaviours; significant overlap between different types of addictions.	Barnes et al. 2015
van der Maas and Nower 2021	United States	Longitudinal	2,176	No	PGSI	Tobacco use, Substance use problems	DSM	384	- Problem gambling scores are linked to suicidal ideation, tobacco use, and substance use problems among military members. - Active duty members participate more in online gambling, lottery, electronic gambling machines, and sports betting.	Davis et al. 2017
Carr et al. 2021	United States	Cross-sectional	176	No	RAD	Substance use, binge eating, hypersexuality	DSM-V, PHQ, GAD-7	176	Individuals receiving opioid agonist therapies had higher rates of co-occurring gambling, binge eating, and hypersexuality symptoms.	Himelhoch et al. 2016
Larsson and Håkansson 2022	Sweden	Case-control	10776 (n = 3,592 GD cases)	Yes (n = 7184)	ICD-10	Mental illness, socio-economic situation	ICD-10, national registries	3,592	Individuals with GD had higher rates of mental illness and poorer socio-economic situations compared to controls.	Edens and Rosenheck 2012
André et al. 2021	Sweden	Cross-sectional	1007	No	GAS, PGSI	Alcohol, illicit drug use/prescription sedatives/strong painkillers	Kessler-6 distress scale.	1007	Increased distress & problem gambling with higher gaming severity. 50% co-occurrence of addictions among addictive gamers.	Langan et al. 2019
Szerman et al. 2023	Spain	Criss-Sectional	116	No	MULTICAGE-CAD-4 and SOGS	SUD, Mood Disorders, ADHD, Impulse-Control Disorders	SDS, BDI-II, STAI, ASRS and CAARS	113	- High co-occurrence of GD with other mental health disorders in a clinical sample. - Calls for reconceptualizing GD as a ‘gambling dual disorder’ due to frequent comorbidity.	van der Maas and Nower 2021
Pagano et al. 2023	United States	Longitudinal	651	No	SOGS	Tobacco use	AUDADIS-IV	102	GD was associated with poor health outcomes and increased tobacco use among those receiving substance use treatment.	Larsson and Håkansson 2022
McGrath et al. 2023	Canada	Longitudinal	10,054	No	PGSI	Substance use, mental health	CCFS-MH	2553	Cannabis use was associated with more severe problem gambling, increased gambling behaviour, and poorer mental health among gamblers.	Hammond et al. 2020
Leino et al. 2023	Norway	Longitudinal registry	140857 (Norwegian patient registry)	No	ICD-10	Substance use disorder	ICD-10	986	− 0.7% of patients diagnosed with both GD and SUD disorder; individuals with SUD had an increased risk of developing gambling disorder, and vice versa, suggesting a strong bidirectional relationship.	Bergamini et al. 2018
Girard et al. 2023	Norway	Longitudinal	5131	No	ICD-110	Substance use disorder	ICD-10	1169	SUD diagnosis more likely precedes Gambling Disorder diagnosis	Kotyuk et al. 2020

Game Addiction Scale (GAS); MINI-International Diagnostic Interview for DSM-IV (MINI-IDIV); Alcohol Use Disorder and Associated Disability Interview Schedule-DSM-IV Version; South Oaks Gambling Screen (SOGS); Materialism Values Scale (MVS); Harvard Department of Psychiatry/National Depression Screening Day Scale (HANDS); Mood Disorder Questionnaire (MDQ); Short Posttraumatic Stress Disorder Rating Interview (SPRINT); National Opinion Research Centre (NORC) DSM-IV NODS-SA; Coping Strategies Inventory (CSI); Difficulties in Emotion Regulation Scale (DERS); Canadian Problem Gambling Index (CPGI); Recognising addictive disorders (RAD); Alcohol Use Disorders Identification Test (AUDIT): Barratt Impulsivity Scale (BIS); Brief Biosocial Gambling Screen (BBGS); Mini International Neuropsychiatric Interview (MINI); Alcohol, Smoking and Substance Involvement Screening Test (ASSIST); Problem Gambling Severity Index (PGSI); Composite International Diagnostic Interview (CIDI) & CAGE; Diagnostic Interview Schedule (DIS-IV for pathological gambling); National Opinion Research Centre DSM-IV Screen for Gambling Problems(NODS); Alcohol Use Disorders Identification Test-Consumption Questions (AUDIT-C); Spanish version of the severity of dependence scale (SDS); Beck’s Depression Inventory (BDI-II); State-Trait Anxiety Inventory (STAI) and the Social Phobia Inventory (SPIN); Adult Self-Report Scale version 1.1 (ASRS-v.1.1) and the Conners’ Adult ADHD Rating Scale (CAARS); PTSD Checklist-Specific Stressor version (PCL-S); Patient Health Questionnaire-4 (PHQ-4); Fagerstrom Test for Nicotine Dependence (FTND); Pittsburgh Sleep Quality Index (PSQI); Bergen Social Media Addiction Scale (BSMAS); Exercise Addiction Inventory (EAI); Massachusetts General Hospital Hairpulling Scale (MGH-HPS); Problematic Internet Use Questionnaire (PIUQ); Problematic Online Gaming Questionnaire Short-Form (POGQ-SF)

#### Studies of patients in treatment for PG substance and alcohol use disorders

Co-occurring disorders such as mood disorders, generalised anxiety disorder (GAD), and post-traumatic stress disorder (PTSD) were common among 71 treatment-seeking pathological gamblers (Soberay et al. 2014). Furthermore, the number of co-occurring disorders was positively related to gambling problem severity at treatment onset and negatively related to psychosocial functioning. Lifetime gambling symptoms were associated with Axis II disorders but not Axis I disorders in a US sample of 402 patients receiving substance use treatment (Cowlishaw and Hakes 2015). While gambling problems were elevated in this population, they may be less common than previously thought. A high prevalence of gambling disorder (46.2%) was reported among methadone maintenance patients (*N* = 185), with lottery tickets being the most common form of gambling. Despite high unemployment rates, participants with GD spent significantly on gambling (Himelhoch et al. 2016). While demographics weren’t linked to GD, those with GD reported engaging in a wider variety of gambling behaviours and were more likely to disclose them to healthcare providers. Patients in opioid maintenance treatment (*N* = 355) were less likely than pathological gamblers to have high comorbidity with psychiatric disorders (Silbernagl et al. 2019). However, individuals with attention-deficit/hyperactivity disorder (ADHD) in childhood and those with adult ADHD had a higher probability of having high co-occurrence with psychiatric disorders. Most patients receiving opioid agonists from an outpatient clinic (*N* = 176), most did not report symptoms beyond drug or tobacco use (Carr et al. 2021). However, 7% to 47% of patients reported some symptoms of other addictive behaviours, such as gambling, binge eating, and hyper-sexuality. Higher impulsivity predicted the presence and/or increased severity of symptoms of drug use, gambling, binge eating, and hyper-sexuality. Higher depression predicted increased severity of drug use and binge eating symptoms, while increased anxiety predicted lower severity of alcohol use and binge eating but higher severity of smoking symptoms. A high rate of comorbid substance dependence was observed in a group of 515 male pathological gamblers receiving treatment, with 88% having a comorbid diagnosis (Mann et al. 2017). The Axis I comorbidity rates were particularly high for nicotine and alcohol dependence among pathological gamblers.

Furthermore, an early age of first gambling experience correlated with greater gambling severity, and first-degree relatives of pathological gamblers were more likely to suffer from alcohol dependence, pathological gambling and suicide attempts. 35% of 288 Veterans seeking treatment in VA outpatient mental health and SUD clinics in the US reported gambling in the past 30 days (Davis et al. 2017). Age, recent binge drinking, and non-partner physical aggression were associated with recent gambling. Among 900 outpatients at mental health centres in the US, comorbidities included schizophrenia and related disorders, bipolar disorder, unipolar depression, and cluster B personality disorders (Bergamini et al. 2018). In 52.1% of the cases, at-risk gambling preceded the onset of a major psychiatric disorder. High-risk gambling in psychiatric patients was four times higher than in community controls, while in substance users, high-risk gambling was twice that of non-users. Among 2,176 adult residents, problem gambling scores for past and current military service members were more than double that of civilian participants (van der Maas and Nower 2021). Notably, the relationship between problem gambling scores and military service was stronger for women than men. Active military service members scored higher on the Problem Gambling Severity Index and showed nearly 20 times the rate of suicidal ideation compared to civilians.

Furthermore, among military service members, problem gambling scores were associated with suicidal ideation, tobacco use, and substance use problems. In a study involving *N* = 140,857 patients in Norway, 23% of GD patients were also diagnosed with SUD, whereas only 0.7% of SUD patients also had GD (Leino et al. 2023). Within the GD patients population, males were more likely to develop SUD in the same year compared to females, but females were more likely to develop SUD a year or more after the onset of GD. Among SUD patients, males were more likely to develop GD in all age categories and periods, except those between 40 and 66 years, whereas the risk was similar for both genders three to four years after the onset of SUD. Finally, common co-occurring addictive behaviours, including compulsive buying, internet use, video gaming, and SUD, primarily tobacco and alcohol, were found among 116 patients with GD in a specialised centre in Spain (Szerman et al. 2023). Half of these patients had ADHD, 30.2% showed moderate or severe depression, and 17.2% had social anxiety disorder.

*In summary*, among outpatients in psychiatric treatment opiate maintenance treatment and veterans, mood disorders, generalised anxiety disorder (GAD), and post-traumatic stress disorder (PTSD), including a history of physical trauma or sexual trauma, are common. Other conditions identified were schizophrenia and related disorders, bipolar disorder, and cluster B personality disorders. Higher impulsivity predicted the presence and/or increased severity of symptoms of drug use, gambling, binge eating, and hyper-sexuality.

### Prospective studies

There are few prospective studies on the comorbidity of pathological gambling with other disorders. A prospective study of 101 pathological gamblers who had recently quit gambling showed that participants with a lifetime drug diagnosis were less likely to maintain at least three months of abstinence (Hodgins and El-Guebaly 2010). Additionally, a lifetime history of mood disorder was associated with a longer time to achieve at least three months of continuous abstinence. Both gambling treatment and a diagnosis of alcohol use disorder were associated with an increased likelihood of relapse after a minimum six-month period of abstinence. In nationally representative US samples collected in 2000–2001 and 2004–2005, involving 33,231 participants, past-year disordered gambling at baseline was associated with an increased likelihood of developing any Axis I psychiatric disorder, mood disorder, bipolar disorder, generalised anxiety disorder, post-traumatic stress disorder, substance use disorder, alcohol use disorder, and alcohol dependence disorders (Chou and Afifi 2011). These associations persisted even after accounting for sociodemographic variables, medical conditions, health-related quality of life, and recent stressful life events. NESARC data (*N* = 10,231) on problem gambling and Axis I psychopathology over two waves (Wave 1: 2000–2001; Wave 2: 2004–2005) was analysed by Pilver et al. (2013), who found that at-risk/problem/pathological gambling (ARPG) in wave 1 was positively associated with the incidence of GAD and SUD. In contrast, low-risk gambling (LRG) was associated with a lower incidence of hypomania. Additionally, older adult gamblers were also at increased risk of developing psychiatric disorders. Longitudinal data from NESARC (*N* = 34,653) were analysed by Parhami et al. (2014), who found that three years after the initial intake interview, individuals who reported any gambling behaviour at baseline had a higher likelihood of developing mood, anxiety or substance use disorders. Multiple specific gambling-related symptoms were associated with comorbid disorders, possibly indicating the interaction of different mechanisms linking gambling disorder with the onset of comorbid psychopathology.

*In summary*, these prospective studies emphasise the long-term impact of GD on the development and persistence of psychiatric comorbidities. Factors such as a history of substance use or mood disorders, age and specific gambling-related symptoms appear to interact with mechanisms that underlie the development of comorbid conditions.

### Affective disorders

Studies investigating the relationship between pathological gambling and mood disorders have been conducted in both general and specific populations.

### Population studies

In general population studies, a large-scale Canadian Community Health Survey (*N* = 10,056) found associations between past-year PG and anxiety or mood disorders, with women showing increased risks for mood disorders (depression, panic attacks, and bipolar disorder), suicidal ideation, psychological distress, and alcohol dependence (Afifi et al. 2010). Similarly, a longitudinal study (*N* = 517) identified that at-risk or PG was associated with new-onset mental disorders such as depression and SUDs, including alcohol dependence and illegal drug (Afifi et al. 2016). Another study of Canadian pathological gamblers in the community (*N* = 105) revealed that those with comorbid depression showed more severe gambling problems, higher rates of childhood trauma, and distinct personality traits (such as higher neuroticism, lower extraversion, agreeableness and conscientiousness) compared with those without depression (Quigley et al. 2015).

### Specific population

A sample of *N* = 158 outpatients with Bipolar disorder had higher pathological gambling scores compared with control participants (Di Nicola et al. 2010), while a Canadian and US study (*N* = 579) of individuals with major depressive disorder (MDD) or bipolar disorder (BD) found no difference in PG prevalence between groups, though males with BD had a higher prevalence of PG (19.5%) compared to females (7.8%) with mood disorders preceded PG in 71% of comorbid cases in this sample (Kennedy et al. 2010). Among psychiatric outpatients in Canada (*N* = 275), PG prevalence was higher in those with lifetime depressive or bipolar disorder compared to the general population, but there is no difference in PG prevalence rates between those with depressive disorders and bipolar disorders (Quilty et al. 2011). Lister et al. (Lister et al. 2015)studied the problem and pathological gamblers (*N* = 150, age 18–80 years) and found that those with a current co-occurring mood disorder were more likely to be female and older and report higher lifetime and past-year gambling severity. Personality factors such as lower social closeness and higher alienation increased the likelihood of being diagnosed with a co-occurring mood disorder. Finally, among 61 patients with GD and comorbid depression found that CBT, treatment reduced gambling scores, depression, and craving, while improving gambling control and depressive symptoms (Linnet et al. 2017).

*In summary* – PG is associated with a high incidence of depression and anxiety disorders. Women with PG are more likely to have mood disorders, high risk of suicidal ideation and attempts, reduced psychological well-being, increased distress, and higher incidence of mania, panic attacks, social phobia, agoraphobia and alcohol dependence.

Table 2 shows studies of comorbidity of Gambling disorder with mood and anxiety disorders.

**Table 2.** GD and associated affective and anxiety disorders.

Study	Country	Study type	Sample Size (n)	Healthy control	Problem/Pathological Gambling Disorder Diagnostic Tool	Associated comorbidity	Comorbidity Diagnostic Tool	No. of Problem / Pathological Gamblers in samples	Key findings	Ref
Afifi et al. 2010	Canada	Cross-sectional	10,056	No	CPGI	Mental disorders, substance use disorders, physical health problems	CIDI DSM-IV	320	- PG in women was associated with lower general health, increased mental disorders, and higher help-seeking behaviour - PG was associated with several physical health conditions including chronic bronchitis, fibromyalgia, and migraines.	Pilver et al. 2013
Kennedy et al. 2010	Canada	Cross-sectional	579	No	CPGI	MDD or BD	MINI	69	MDD (12.5%) & BD (12.3%) have similar problem gambling rates. Males in BD group have higher rates than females.	Quigley et al. 2015
Di Nicola et al. 2010	Italy	Case-control	158	Yes (n = 200)	SOGS	BD, other behavioural addictions (compulsive shopping, sexual addiction, Internet addiction, work addiction, physical exercise addiction)	DSM-IV for SCID-I & II, CBS, SAST, IAD, WART, EAI, BIS for other behavioural addictions	Not specified for gambling alone; 33% of bipolar patients had at least one behavioural addiction	Bipolar patients had a higher prevalence of behavioural addictions (33%) than controls (13%), with elevated scores in pathological gambling, compulsive buying, sexual addiction, and work addiction. These patients showed lower self-directness and cooperativeness, but higher impulsivity. Behavioural addictions were linked to increased impulsivity and character immaturity.	Afifi et al. 2016
Quigley et al 2011	Canada	Longitudinal	275	No	CPGI, SOGS	Mood disorders (Major Depressive and Bipolar Disorder)	SCID-I/P	Not specified	GD is more prevalent in individuals with mood disorders and there is a concurrent association between the symptoms of both, there is no direct longitudinal association between these pathologies	Di Nicola et al. 2010
Giddens et al. 2012	USA	Cross-sectional	43,093 (n = 4,515 with AD; n = 36,334 without AD)	No	DSM-IV	Axis I disorder	AUDADIS-IV	24 (0.46%)	Pathological gambling severity is associated with co-occurring psychiatric disorders, especially in individuals with anxiety disorders.	Edgerton et al. 2018
Quigley et al. 2015	Canada	Cross-sectional	105	No	PGSI	Major depression	PHQ-9	105	Problem gamblers (32.4%) with major depression had more severe gambling issues, a history of childhood abuse, poor family functioning, and different personality traits.	Di Nicola et al. 2010
Lister et al. 2015	Canada	Cross-sectional	150	No	NODS, GMQ	Mood Disorders	SCID-I/P	150	Mood disorders frequently co-occur with problem and pathological gambling, and they are associated with greater gambling severity.	Kennedy et al. 2010
Cartmill et al. 2015	Australia	Cross-sectional	142	No	CPGSI	Association and dissociation	DASS, DQ	60	Anxiety and dissociation predict problem gambling behaviour across gambling modalities.	Wirkus et al. 2024
Afifi et al. 2016	Canada	Longitudinal	679	No	CPGI	Mental and SUD	CIDI-SF, DSM-IV	At-risk or PG, n = 215, 31.5%	Young adults with at-risk or problem gambling were more likely to develop depression, alcohol dependence, and illegal drug use over five years. Conversely, only illegal drug use predicted future problem gambling.	Parhami et al. 2014
Cowlishaw et al. 2016	USA	Cross sectional (NESARC)	3,007	No	DSM-IV	Mood problems, anxiety, depression, social phobia, dysthymia	AUDADIS-IV	Lifetime: 93 (3.1% of 3,007), Past-year: 42 (1.4% of 3,007)	-Lifetime PG rates: 3.1% (depression) to 5.4% (social phobia). Past-year rates: 0.9% (dysthymia) to 2.4% (social phobia). 8.9% had any gambling problems. Gambling problems predicted interpersonal issues, financial difficulties, and marijuana use, but not alcohol use, mental/physical health, or healthcare utilisation.	Black et al. 2021
Jauregui et al. 2016	Spain	Comparative study	328	Yes (n = 204)	SOGS	Depressive and anxious symptomatology	MCQ-30; SA-45	124	Results showed that pathological gamblers had higher levels of depressive and anxious symptomatology.	Kim et al. 2024
Chinneck et al. 2016	Canada	Longitudinal	Wave (I = 679; II = 624; III = 578; IV = 530)	No	PGSI, CPGI, SOGS	Depression	CES-D, CIDI-SF	Wave (I = 601; II = 566; III = 529; IV = 487)	Depressive and PG symptoms in emerging adults were positively correlated, but neither disorder was a risk factor for the other, and their co-occurrence may be better explained by a common underlying factor such as substance abuse.	Lister et al. 2015
Linnet et al. 2017	Denmark		136	No	SOGS	Depression	MDI, SCID-I	61	Treatment significantly reduced SOGS and MDI scores, craving, and improved gambling control. Comorbid depression affected MDI scores and interacted with treatment outcome to reduce depressive symptoms.	Quilty et al. 2011
Rodriguez-Monguio et al. 2017	USA	Retrospective database (APCD)	869	No	ICD-9-CM codes	Anxiety, mood, SUD, depressive disorders	AUDADIS-IV	869	Pathological gambling comorbid with mental health and substance use disorders. Health-care services provision varies by clinician specialty. Integrative treatment is crucial.	Cartmill et al. 2015
Edgerton et al. 2018	Canada	Longitudinal (4 waves over 5 years)	679	No	PGSI	Depression	CES-D, DSM-III R, NEO-FFI, BIS-11	517	Depression and problem gambling symptoms in emerging adults followed five distinct trajectories, but there was no evidence of reciprocal growth between the two, suggesting a common underlying factor such as substance abuse.	Linnet et al. 2017
Black et al. 2021	USA	Longitudinal (5 year follow up)	105	No	DSM-IV and SOGS, NODS	Mood disorders, anxiety disorders, substance use disorders, personality disorders	MINI Axis-I disorder, SIDP-IV for personality disorder	105	At 5-year follow-up, 44% recovered from pathological gambling. Mood and anxiety disorders at baseline predicted continued gambling, while substance use and personality disorders did not, and younger age predicted continued gambling.	Chinneck et al. 2016
Sundqvist and Wennberg 2022	Sweden	Case-control	1876 (case, n = 399)	Yes (n = 1477)	PGSI, SOGS	Anxiety and Panic Disorder, Social Phobia, GAD, PTSD	MINI DSM IV TR	Not reported	All anxiety disorders were linked to problem gambling, especially social phobia. Strongest associations were in participants under 25, females, and middle SES. Participants under 25 had a threefold higher risk of GAD.	Giddens et al. 2012
Wullinger et al. 2023	Germany	Longitudinal one-armed cohort study	123	No	DSM-5	Affective disorders, anxiety disorders	DSM-5, CIDI	123	GD with and without psychiatric comorbidities, benefit from outpatient gambling care. Psychiatric comorbidity, especially comorbid anxiety disorders, seems to be negatively associated with the course of GD within outpatient gambling care.	Jauregui et al. 2016
Wirkus et al. 2024	Germany	Longitudinal	607	No	DSM V	SUD, Impulsivity, mental disorder (depression, anxiety), stress	CIDI, UPPS-P, TAS-26, ERQ, BSI-18, PSS-10	125	Impulsivity, emotional difficulties, stress, and comorbid mental disorders are associated with and predict gambling disorder in online sports bettors.	Cowlishaw et al. 2016
Kim et al. 2024	Canada	Longitudinal	4121	No	PGSI	Depression, Psychosis, OCD, PTSD	ASSIST, CIDI, NEO PIR	Not mentioned	Most people showed a decrease in both gambling and addictions over time. No substitution of addictions was observed.	Sundqvist and Wennberg 2022

Composite International Diagnostic Interview (CIDI DSM-IV); Canadian Problem Gambling Index (CPGI); Major Depressive Disorder (MDD); Bipolar Disorder (BD); Mini International Neuropsychiatric Interview (MINI); South Oaks Gambling Screen (SOGS); Structured Clinical Interview for DSM-IV Axis I and Axis II Disorders (SCID I and II); Compulsive Buying Scale (CBS); Sexual Addiction Screening Test (SAST); Internet Addiction Disorder (IAD); Work Addiction Risk Test (WART); Exercise Addiction Inventory (EAI); Barratt Impulsiveness Scale 11 (BIS-11); Alcohol Use Disorder and Associated Disability Interview Schedule-DSM-IV Version (AUDADIS-IV); Structured Clinical Interview for DSM-IV-TR Axis I Disorders, Research Version, Patient Edition (SCID-I/P); Gambling Motives Questionnaire (GMQ); National Opinion Research Centre DSM Screen for Gambling Problems (NODS); Physical Health Questionnaires (PHQ); Depression, Anxiety and Stress Short-Scale (DASS); Dissociation Questionnaire (DQ); Canadian Problem Gambling Severity Index (CPGSI); Subscales of Anxiety and Depression of the Symptom Assessment-45 Questionnaire (SA-45); Metacognition Questionnaire 30 (MCQ-30); Centre for Epidemiological Studies Depression Scale (CES-D); NEO Five Factor Inventory (NEO-FFI); Generalised Anxiety Disorder (GAD); Major Depression Inventory (MDI); Urgency-Premeditation-Perseverance-Sensation Seeking-Positive Urgency (UPPS-P); Toronto Alexithymia Scale (TAS-26); Emotion Regulation Questionnaire (ERQ); Brief Symptom Inventory (BSI-18); Perceived Stress Scale (PSS-10); Problem and Pathological Gambling Measure (PPGM); NEO Personality Inventory-Revised (NEO PIR).

### Prospective studies

Chinneck et al. (Chinneck et al. 2016) and Edgerton et al. (Edgerton et al. 2018) analysed data from the Manitoba Longitudinal Study of Young Adults (age 18–20). Positive correlations were shown between depressive and PG symptoms, but neither predicted the other Chinneck et al. (Chinneck et al. 2016). Furthermore, there was no reciprocal growth in PG and depression in a study of 679 adults over 5 years (Edgerton et al. 2018). A study of 57 younger adults (18–40 years) and 48 older adults (over 60) with PG over 31 months found that anxiety disorders, mood disorders and impulse control disorders showed the highest problem severity rates during follow-up (Black et al. 2021). More severe depression or PTSD correlated with increased gambling activity. In older adults, more severe agoraphobia and social phobia were associated with decreased gambling activity. For younger subjects, greater severity of SUD, alcohol use disorder, or compulsive computer use correlated with reduced gambling activity.

*In summary*, impulsivity, depression and trauma can be predictors of gambling disorders, but the evidence for reciprocal relationships between mood disorders and gambling disorders is mixed.

### Anxiety disorders

Studies on anxiety disorders and GD have been conducted in both general population samples and specific populations.

### Population studies

An analysis of NESARC data (*N* = 43,093) showed that increased pathological gambling severity was associated with Axes I and II psychopathology in groups with and without anxiety disorders (Giddens et al. 2012). Another NESARC-based study (*N* = 3007) found lifetime pathological gambling rates ranged from 3.1% for depression to 5.4% for social anxiety disorder, with 8.9% of respondents reporting a history of gambling issues when considering at-risk gambling (Cowlishaw et al. 2016). Similarly, a Swedish longitudinal gambling study cohort (*N* = 1,876) reported associations between all anxiety disorders (Panic Disorders, Social Anxiety Disorder, Generalised Anxiety Disorder) and PG, with Social Anxiety showing the strongest links, particularly in younger individuals, females, and middle socioeconomic status (Sundqvist and Wennberg 2022). The RIGAB longitudinal study (*N* = 607) identified predictors of GD severity, such as high impulsivity, alcohol and tobacco use, frequent betting, and substantial financial losses, among online sports bettors (Wirkus et al. 2024). Additionally, a Canadian longitudinal study (*N* = 4121) found a general decrease over time, suggesting recovery from gambling problems aligns with reductions in overall addiction behaviours (Kim et al. 2024).

### Specific populations

In studies focusing on specific populations, Australian gamblers (*N* = 142) demonstrated that anxiety and dissociation independently and together predicted pathological gambling (Cartmill et al. 2015). Male pathological gamblers (*N* = 124) showed greater emotion regulation difficulties and higher levels of anxiety, depression, and drug abuse than non-gambler males (Jauregui et al. 2016). High rates of comorbid disorders: anxiety disorders (28%), mood disorders (26%), and substance use disorders (18%) were reported among *N* = 869 ­primarily male treatment-seeking pathological gamblers (Rodriguez-Monguio et al. 2017). In a study of gamblers across German outpatient addiction care facilities (*N* = 123), those with comorbid anxiety disorders showed less improvement in GD severity compared to those without anxiety disorders, with anxiety often preceding the development of GD (Wullinger et al. 2023).

*In summary*, although studies indicated strong co-morbidity of anxiety disorders and PG, there is evidence that pathological gambling severity and psychopathology were stronger in participants without anxiety disorders and that participants with comorbid anxiety disorders showed less improvement in GD severity compared to those without anxiety disorders.

## Discussion

There is a high comorbidity of problematic and pathological gambling with other mental health disorders, particularly substance and alcohol use and mood disorders. Frequent online gambling and cannabis use are linked with greater gambling severity, suggesting a need for comprehensive treatment approaches (McGrath et al. 2023). There are reciprocal relationships between PG and comorbid conditions. In some cases, gambling precedes the start of the comorbid disorder, while in other instances, it is the comorbid condition that has the early onset. Prospective studies have identified substance abuse, dependence and behavioural addictions as strong predictors of GD development.

Additionally, these studies demonstrated that mood disorders can also predict the development of GD. Negative experiences in early childhood, including abuse and trauma, seem to be associated with the subsequent development of GD (Felsher et al. 2010). Lorains et al. (2011), in their meta-analysis, reported high comorbidity rates of PG with nicotine dependence, substance use disorder, mood disorders, and anxiety disorders. They noted moderate heterogeneity across studies, indicating that the rate estimates do not consistently converge around a single population figure. Dowling et al. (Dowling et al. 2015), in their meta-analysis, found high rates of comorbid current and lifetime Axis I disorders among pathological gamblers, including mood, alcohol use, anxiety, and non-alcohol substance use disorders. Recently, Grant and Chamberlain (Grant and Chamberlain 2020) estimated that 28–50% of individuals with PG have lifetime substance use disorders, with anxiety and mood disorders being more common. Elevated rates of these disorders may also be linked to co-occurring personality disorders, mainly cluster B (antisocial, borderline, avoidant, and paranoid) (Grant et al. 2005) or attention-deficit hyperactivity disorder (ADHD) (Grall-Bronnec et al. 2011). ADHD and antisocial and borderline personality disorders have been highly associated with impulsivity.

Cross-sectional studies showed sex differences in gambling disorder, with women facing greater psychiatric comorbidity and socioeconomic vulnerability. Women with GD are more likely to have mood disorders and a higher risk of suicidal ideation and attempts, mania, anxiety and alcohol dependence (Afifi et al. 2010). Among treatment-seeking pathological gamblers, including patients in opiate maintenance treatment and veterans, several comorbid conditions are common, such as mood disorders, GAD, PTSD, and a history of physical or sexual trauma. Additionally, other conditions identified were schizophrenia and related disorders, bipolar disorder, and cluster B personality disorders.

Prospective studies have identified substance abuse, dependence, behavioural addictions and early negative childhood experiences, including abuse and trauma, are predictors of future GD (Felsher et al. 2010). Although there is a strong co-morbidity of anxiety disorders and PG, anxiety is not related to different levels of gambling, suggesting that patients without anxiety disorders display stronger pathology. Furthermore, participants with comorbid anxiety disorders showed less improvement in GD severity compared to those without anxiety disorders (Wullinger et al. 2023). These studies emphasise the need for integrated treatment approaches addressing both gambling behaviour and associated substance use mood and anxiety disorders.

### Pharmacological treatment and CBT: Conventional to novel therapeutic approach

The majority of evidence supporting treatments for gambling disorder is based on psychological interventions, especially CBT, which has been shown to be effective gambling disorder and various comorbidities (see Dowling (Dowling et al. 2015) for a review). A recent meta-analysis found that psychological interventions, particularly face-to-face CBT, are effective in treating GD and significantly reducing and improving quality of life (Eriksen et al. 2023). Pharmacological interventions for GD have shown varying degrees of efficacy, with some evidence supporting the use of opioid antagonists, particularly naltrexone, although this is based on an opinion rather than on empirical evidence. Systematic reviews by Grant et al. (Grant et al. 2014) and Lupi et al. (Lupi et al. 2014) suggest that pharmacological treatments such as opioid antagonists (e.g., Naltrexone and Nalmefene), glutamatergic agents (N-acetyl cysteine, memantine, amantadine) antidepressants (paroxetine, fluvoxamine, sertraline, escitalopram) and mood stabilisers (lithium carbonate and topiramate) show promise in managing symptoms of pathological gambling, though the evidence remains limited by small sample sizes and methodological variability. Additionally, a review on lithium treatment for gambling disorder and bipolar disorder by Di Nicola (Di Nicola et al. 2014) concluded that ‘only a few clinical trials are available and the population is limited; therefore no conclusive evidence can be inferred’. While selective serotonin reuptake inhibitors (SSRI) have produced mixed results, they may be beneficial in cases with comorbid depression or anxiety disorders, as shown in studies of paroxetine (Kim et al. 2002) and sertraline (Saiz-Ruiz et al. 2005). Lithium has been found to be particularly effective in treating GD in patients with bipolar spectrum disorders, with one controlled trial showing a significant reduction in both gambling behaviour and affective symptoms (Hollander et al. 2005). Recent advances in GD with comorbid psychiatric conditions include digital interventions, such as mobile apps, which have reduced gambling behaviour (Hodgins et al. 2019). Neuromodulation techniques such as repeated transcranial magnetic stimulation (rTMS) can reduce gambling reinforcement in non-comorbid men with PG (Zack et al. 2016). Integrated models treating GD and comorbidities together offer superior results, while telehealth has expanded access and maintained effectiveness, especially during COVID-19 (Smith et al. 2022).

### Limitations

This narrative review is limited by its lack of rigorous methodology compared to systematic reviews and meta-analyses. The potential selection bias from including only English-language studies, and the high heterogeneity of study designs and populations makes it challenging to generalise findings and draw consistent conclusions. The review also cannot establish causal relationships between GD and comorbidities, as these interactions are often complex and bidirectional.

## Conclusions

Over the past 15 years, significant progress has been made in treating GD and its psychiatric comorbidities, with evidence highlighting the reciprocal relationships between GD and conditions like substance use, mood and anxiety disorders. CBT remains the standard, with strong evidence supporting its effectiveness. Pharmacological options, such as naltrexone and N-acetylcysteine, show promise for specific cases but require further research due to limited evidence. Emerging therapies, including neuromodulation and digital interventions, offer innovative options, while integrated models addressing both GD and comorbidities provide superior outcomes. Telehealth has expanded treatment accessibility, particularly during the COVID-19 pandemic; pharmacological and novel therapies are essential to improving outcomes, with future research focusing on personalised and accessible intervention.
